# Treatment of distal tibial fractures with the Ilizarov external fixator - a prospective observational study in 39 consecutive patients

**DOI:** 10.1186/1471-2474-14-30

**Published:** 2013-01-17

**Authors:** Telmo Ramos, Jón Karlsson, Bengt I Eriksson, Lars Nistor

**Affiliations:** 1 Department of Orthopaedics, Central Hospital (Kärnsjukhuset), Skövde, SE-541 85, Sweden; 2 Department of Orthopaedics, Sahlgrenska University Hospital, Sahlgrenska Academy at Gothenburg University, Mölndal, SE-431 80, Sweden

**Keywords:** Distal tibial fractures, Ilizarov method, External fixation

## Abstract

**Background:**

The management of displaced distal tibial fractures is still controversial. The different internal fixation techniques are often burdened by relatively high complication rates. Minimally invasive techniques with ring fixators have been introduced as an alternative allowing immediate reduction and stabilization, avoiding a staged protocol. The aim of this prospective study was to analyze the clinical and radiographic outcome the Ilizarov technique in patients with distal metaphyseal tibial fractures, with or without intra-articular involvement.

**Methods:**

Thirty-nine consecutive patients with isolated fractures treated with the Ilizarov technique were followed prospectively for one year. Depending on the type of fracture, 4 or 5 rings were used, in some cases with additional foot extension. Unrestricted weight-bearing was allowed in all cases. Pre- and post-operatively conventional radiographs, post-operative pain assessment and complications were evaluated. The function was evaluated clinically and with self-appraisal protocols: EQ-5D, NHP and FAOS.

**Results:**

No patient developed compartment syndrome or deep venous thrombosis. Pin infections were frequent, but they were mostly superficial and were treated with antibiotics and/or the removal of isolated pins. Two patients required debridement. One of them had a deep infection and developed a residual deformity which was corrected and healed after re-operation. Another patient had a severe residual deformity. The fixator was removed after a median period of 16 weeks (range 11–30). The radiological results were poor in 5 patients but the overall self-appraisal showed satisfactory results in 36 patients.

**Conclusions:**

The Ilizarov method allowed early definitive treatment with a low complication rate and a good clinical outcome.

## Background

When treating distal tibial fractures, the goal is to achieve normal axial alignment and to reduce articular displacement if present, thereby regaining a stable, mobile and painless joint, while avoiding infections and wound complications [1].

The treatment of these fractures is challenging [2-5]. It is often difficult to assess the potential risk of surgical complications because of the variations in the clinical findings. Sometimes the injury can be more serious than initially expected, even in patients without articular involvement. One main reason is probably the underestimation of the soft-tissue injuries, not addressed in the fracture classification [6].

In intra-articular fractures, the sequential management principles outlined by Rüedi and Allgöwer [7] are generally accepted. The aim of the first step is to preserve length with a joint-bridging fixator or a fibular plate and, when the soft-tissue injuries permit, the definitive step is traditionally performed with screws and plates [8-11]. In less comminuted intra-articular fractures (Rüedi-Allgöwer types I and II), McFerran et al. reported a 54% risk of major complications [4]. There are studies indicating that it is possible to reduce the number and severity of complications using a staged protocol [9,12-15].

Even if extra-articular fractures are expected to be associated with less risk of treatment complications than intra-articular fractures, the proximity to the ankle and the vulnerable soft-tissue in the distal region increases the risk of complications compared with midshaft tibial fractures [16,17].

The union rate in these fractures is still reported as 2.4% and the malunion rate as 14.3% independent of the treatment approach [18]. The current knowledge indicates that is essential to consider the risk of soft-tissue complications and fracture malalignment when selecting the method of fixation [18,19].

When it comes to intra-articular distal tibial fractures, there are reports of the use of an initial joint-bridging fixator, subsequently converted to a non-bridging device, showing lower complication rates compared with internal fixation [20-23]. The use of primary circular fixators (in accordance with Ilizarov principles), with or without minimal internal osteosynthesis, has also been reported to reduce the complication rate in these fractures [24-28].

With the Ilizarov technique, it is always possible to treat the patients with an immediate one stage procedure [29,30], since reduction is less invasive, with minimal soft-tissue exposure and blood loss. If needed, this fixator also allows for adjustment of the alignment and for compression/distraction both during and after surgery. An additional advantage is that the fixation is stable enough to allow early weight-bearing [31,32].

At our department at the Skaraborg Central Hospital (Kärnsjukhuset) in Skövde, a referral trauma centre for a population of approximately 280.000 inhabitants, the Ilizarov external fixator was gradually introduced for complex distal tibial fractures in 2002 and since 2005, it has been the preferred treatment for displaced distal tibial fractures of all types where the soft tissues were impaired or at risk. The aim of this prospective study was to analyse the clinical and radiographic outcome using the Ilizarov technique in consecutive patients with distal metaphyseal tibial fractures, with or without intra-articular involvement.

## Methods

The selection criteria in this study were as follows: patients aged 18–75 years, with displaced distal metaphyseal (defined by the Heim's square) tibial fractures with an angulation of more than 10 degrees in any plane and intra-articular fractures were included if the incongruence of the articular surface was more than 2 mm. Only patients with isolated fractures, without other disorders affecting gait, who were able to understand and follow instructions in Swedish, were enrolled after written informed consent to participate in the study was obtained.

The fractures were classified according to the AO classification [33]. Plafond fractures were also classified according to Rüedi and Allgöwer [7] and open fractures were classified according to Gustilo [34,35]. The soft-tissue damage was graded according to the Tscherne classification for closed fractures [36].

The operations were performed without a tourniquet and without any traction table. Arthroscopy or arthrotomies were not used. Biplane fluoroscopy was used during reduction, pin insertion and assembly of the frame. The fractures were reduced with traction and manual external pressure. If this did not lead to acceptable anatomical repositioning, the joint surfaces were reconstructed with percutaneously inserted elevators and/or a reduction forceps and/or wires with olives. The proximal ring was placed at the level of the fibular head. Additional stability was achieved using extra wires parallel to the articular surface with posts fixed on the distal ring (drop-wire technique). The syndesmosis and malleolar fragments could be stabilised with olive wires fixed to the ring on the lateral side or the medial side. All the wires were assembled and tensioned to a minimum of 120 kg. To achieve further stability of the system and to allow for unrestricted weight-bearing, additional rings were used in the tibia. Steel rings connected with steel rods were used (Smith & Nephew, Memphis, Tennessee, USA). Bone grafts were not used. All the operations were supervised or performed by one senior surgeon (TR).

Cloxacillin (2 g) was used as prophylaxis starting pre-operatively and continued with another two doses within 24 hours. Low-molecular heparin prophylaxis was given from the day of admission until 10 days after leaving the hospital. During the first 24 hours after surgery all patients had a postoperative continuous analgesia (PCA) pump with morphine/ketobemidon.

The “Kurgan protocol” [37] was used for postoperative dressing and the Checketts-Otterburns classification [38] was used to report pin infections.

Physiotherapy was started immediately postoperatively to maintain knee and ankle motion and the patients were allowed to start unrestricted weight-bearing.

The fractures were regarded as healed when antero-posterior and lateral radiographs showed a bridging callus in three of four cortices and/or the fracture was stable when stressed manually and the patients were able to walk without pain after the connecting rods had been removed.

The patients were followed clinically and radiographically after 2, 4, 8 and 12 weeks and one year. Additional clinical and radiographic assessments were made when necessary to evaluate fracture healing. The clinical one-year outcome, including the range of motion of the ankle, was assessed by an independent physiotherapist.

Pain and patient satisfaction were registered (VAS 100 mm) at four and 12 weeks and at the one year follow-up. The Swedish versions of the EuroQol [39] and the Nottingham Health Profile (NHP) [40,41] were used for patient self-appraisals at the same time intervals. The FAOS [42] questionnaire was added to the follow-up between 1–5 years postoperatively, Pain (VAS), EQ-5D and NHP questionnaires were repeated if the observation period exceeded one year.

Marsh and coworkers [43] modification of the criteria defined by Burwell and Charnley [44] was used to evaluate the articular reduction. This was done by one of the authors (TR) and separately by an independent surgeon for reliability. In the event of different judgements, the final evaluation was made by consensus.

### Statistical analysis

Descriptive statistics as median and range was calculated. As the number of patients in the subgroups was small and as several of the variables were of ordinal data type, we decided to use non-parametric tests for statistical analysis. All the statistical tests were two-sided. For comparisons within the group we used Wilcoxon’s test and between the groups Mann–Whitney test. PASW statistics (SPSS) version 18 was used for all statistical analysis.

The study was approved by the regional ethical review board at Sahlgrenska University Hospital in Gothenburg (ID. 400–04).

## Results

Between January 2005 and December 2010, 39 consecutive patients admitted to the emergency department fulfilled the inclusion criteria. Their median age was 50 years (range 20–70), 27 were women and 12 men. Nine patients were smokers. Individual data on the fractures are given in Table 1. The cause of the injury was falls in 25 patients, motor-vehicle accidents in 5, work accidents in 3, football accidents in 3 and riding accidents in 1 patient.

**Table 1:** Injury type, treatment and pin infections in 39 patients with distal tibia fractures treated with the Ilizarov application

**Case**	**Age**	**Injury**	**Energy**	**AO**	**Rüedi-Allgöwer**	**Gustilo**	**Tscherne**	**Extension from the joint/mm**	**Bone defect/mm**	**Ilizarov rings**	**Foot extension**	**Pin infection**	**Checketts-Otterbuns**
1	20	fall	low	A2				117		4		2	III + III
2	62	fall	low	C1	I			109		4		1	II
3	57	riding	high	C1	I	2		58		4		0	
4	59	fall	low	A2			1	136		4		1	II
5	39	traffic	high	C2	II			66		4	yes	0	
6	55	work	low	A2				152		4		0	
7	56	fall	low	A2				118		4		1	II
8	39	fall	low	C1	II			122		4		2	III
9	33	traffic	high	A3		1		75		4		1	II
10	59	fall	low	C1	I			85		4		2	III + III
11	70	fall	low	A2				172		4		1	II
12	61	work	low	C1	I		1	123		4		1	II
13	58	fall	low	A3			1	126		4		0	
14	50	fall	high	C2	II	1		83	12 × 5	4		1	III
15	46	fall	low	A1		1		107		4		0	
16	43	fall	low	C1	I			168		4		3	II + II + III
17	29	traffic	high	A3		1		241		5		1	II
18	50	trafic	high	C2	II	2		61		4	yes	1	II
19	46	fall	low	C1	I			176		4		3	II + III + VI
20	39	fall	low	A2				172		4		1	II
21	63	fall	low	A2				127		4		0	
22	42	fall	low	A2		1		195		5		2	II + II
23	21	fall	low	A2				93		4		0	
24	42	fall	low	C3	III	1		59		4		0	
25	66	fall	high	C3	III			76	16 × 15	3	yes	0	
26	51	fall	low	C1	I	2		147		4		1	III
27	62	fall	low	C3	III			143		4	yes	2	II + III
28	54	traffic	high	A2				176		4		2	II + II
29	54	fall	low	C1	I			158		4		2	II + III
30	68	fall	low	A2				167		4		3	II + III + IV
31	56	fall	low	A1				133		4		2	II + III
32	43	skiing	low	A3				250		4	yes	2	II + III
33	44	skiing	low	A1				152		4		2	II + III
34	48	fall	low	C1	I			127		4		2	II + III
35	56	traffic	high	C1	I		1	137		4		1	III
36	24	fall	high	C1	II			147	8 × 5	4	yes	1	III
37	42	fall	low	A1				273		4		0	
38	70	fall	low	A3				110		4	yes	0	
39	49	fall	low	A1				143		4		0	

Five patients had type A1 fractures, eleven had A2, five had A3, twelve had C1, three C2 and three C3. Of 18 patients with C-type fractures, ten had Rüedi-Allgöwer I, five had type II, and three type III. Thirty-seven fractures had a diaphyseal extension, which extended more than 10 cm above the articular surface in 30 patients. Thirty patients had closed fractures and nine patients had open fractures, six Gustilo I and three Gustilo II. The soft-tissue damage in the closed fractures was graded according to the Tscherne classification as grade 0 in twenty-six and grade I in four patients. In summary, almost all the fractures included in this study had at least one factor which could increase the risk of complication under the treatment, such as high-energy trauma, communition, soft-tissue injury or long fracture line.

The majority of the patients underwent surgery on the day of admission or within two days. In three patients, the operation was delayed for another 2–3 days because of a high load at the operation department. In the majority of cases four rings, connected with steel rods, were used. In four patients with more comminuted fractures, a foot fixation with trans-calcaneal and trans-metatarsal wire fixation without hinges (foot extension), was added to the construction. Six minor re-operations were performed under general anaesthesia because of the re-insertion of wires after breakage or to improve fracture alignment. The median duration of surgery, which includes assembling the frame and dressings, was shorter for the extra-articular fractures, 152 min (range 50–224), compared with intra-articular fractures, 165 min (range 72–314). Patients with a foot extension had this fixation removed after 4–6 weeks and the Ilizarov fixator was removed under local anaesthesia after 17 weeks (range 12–30) in the extra-articular fractures and 15 weeks (range 11–22) in the intra-articular fractures.

The total amount of morphine/ketobemidon (PCA pump) varied between 0 and 141 mg (median 43 mg). The demand for additional analgesics was low. All the patients were discharged directly to their homes after a median of 5 days post-operatively (range 2–10) when they were able to walk with crutches and independently climb stairs.

The timing of surgery and postoperative care are shown in Table 2, where the fractures have been divided in two groups, extra- and intra-articular. The differences between the groups are not statistically significant.

**Table 2:** Timing of the treatment

	**Extra-articular Factures (n = 21)**		**Intra-articular Fractures (n = 18)**	
	**Median**	**Range**	**Median**	**Range**
Surgery delay (days)	2	(0–24)	1	(0–5)
Operation time (min)	152	(50–224)	165	(72–314)
Hospital stay (days)	5	(3–10)	5	(2–7)
External fixation (weeks)	17	(12–30)	15	(11–22)

A total of 157 rings, with 551 wires, were used, constituting 1.102 potential pin-infection sites.

One patient (# 30), with an A2 fracture, had a pin tract infection grade 4, which was successfully treated with soft-tissue curettage. After the fixator had been removed, in one patient (# 19), with a C1 fracture, a deep S.aureus infection was diagnosed in an area with a previous pin scar. The lesion was treated with curettage, but there was a progressive loss of reduction. This was treated successfully with stabilisation and progressive correction with a new Ilizarov external fixator application for a period of 23 weeks combined with antibiotic therapy (clindamycin) for 12 weeks.

Forty-two minor pin site infections were observed: 24 Checketts-Otterburns II in 19 patients which were treated with short-term antibiotics, and 18 Checketts-Otterburns III in 15 patients treated with the removal of the offending wire (Table 1).

No patients developed compartment syndrome or deep vein thrombosis.

Compared with the uninjured, 3 patients had reduced dorsiflexion of more than 10° (14°, 15°, 16°). The plantar flexion was reduced by more than 10° in 7 patients in the A group (median 15°, range 12°-23°) and in 9 patients in the C group (median 22°, range 18°- 33°). The results are shown in Table 3.

**Table 3:** Range of motion at one year (median and range)

	**Extra-articular Fractures (n = 21)**				**Intra-articular Fractures (n = 18)**			
	**Uninjured**		**Injured**		**Uninjured**		**Injured**	
Ankle dorsiflexion	20°	(0-50°)	18°	(0-50°)	21°	(10-29°)	17°	(4-26°)
Ankle plantarflexion	38°	(12-55°)	30°	(11-52°)	33°	(16-56°)	19°	(4-48°)

With the exception of one patient (#19), all the fractures healed when the fixator was removed (see above). According to the radiological findings using the modified Burwell and Charnley classification, 13 patients were rated as good, 21 as fair and 5 had poor results at the one-year follow-up. The radiological results are shown together with self-appraisals (FAOS and VAS-pain) in Table 4. In Table 5, the radiological results in patients with at least one parameter classified as poor and/or pin tract infection at one year are shown, together with FAOS and VAS-pain.

**Table 4:** The radiological outcome in the Burwell and Charnley classification analyzed with FAOS and VAS satisfaction in both groups at the one-year control

**Intra-articular fractures**															
**Case**	**AO**	**Central fragment/mm**	**Talar subluxation/mm**	**Mortise widening (mm)**	**Varus**	**Valgus**	**Anterior**	**Posterior**	**Burwell & Charnley**	**FAOS Pain**	**FAOS Symptom**	**FAOS ADL**	**FAOS Sport**	**FAOS Qol**	**VAS Satisfaction/mm**
2	C1	<2	<0.5	<0.5	9			5	fair						0
3	C1	<2	<0.5	<0.5				7	fair	100	93	100	100	94	7
5	C2	<2	<0.5	<0.5	8			4	fair	72	21	93	35	38	18
8	C1	<2	<0.5	<0.5	3				good	100	100	100	100	100	38
10	C1	<2	<0.5	<0.5		10	10		poor						8
12	C1	<2	<0.5	<0.5	11		2		poor	72	89	90	60	75	31
14	C2	<2	<0.5	<0.5	8				fair	92	86	100	100	94	10
16	C1	<2	<0.5	<0.5					good	47	64	68	20	44	27
18	C2	<2	<0.5	<0.5					good	61	75	85	35	56	7
19	C1	<2	<0.5	<0.5		3		7	fair	33	61	85	40	63	49
24	C3	<2	<0.5	<0.5		9	6		fair	33	14	71	0	19	54
25	C3	<2	<0.5	<0.5		12		3	poor	31	21	60	5	0	47
26	C1	<2	<0.5	<0.5	6		3		fair	39	64	72	5	75	76
27	C3	9	6	5		16		1	poor	94	57	97	70	63	7
29	C1	<2	<0.5	<0.5			2		good	94	93	97	85	88	7
34	C1	<2	<0.5	<0.5	8		2		fair	67	54	85	40	31	41
35	C1	<2	<0.5	<0.5	1			4	good	100	100	100	100	94	0
36	C1	<2	<0.5	<0.5					good	89	54	100	70	56	2
**Extra-articular fractures**															
**Case**					**Varus**	**Valgus**	**Anterior**	**Posterior**	**Burwell & Charnley**	**FAOS Pain**	**FAOS Symptom**	**FAOS ADL**	**FAOS Sport**	**FAOS Qol**	**VAS Satisfaction/mm**
1	A2						7		fair						0
4	A2					4		8	fair	100	100	100	100	100	18
6	A2					8	1		fair	83	68	93	70	75	0
7	A2							8	fair						26
9	A3					5	5		fair						0
11	A2					2			good	100	100	97	90	81	0
13	A3					5	4		fair	97	86	96	100	88	38
15	A1				5		8		fair	100	86	100	100	100	0
17	A3					4	2		fair	100	93	99	95	100	0
20	A2								good						54
21	A2				6		4		fair	100	100	100	100	100	7
22	A2					1		2	good	67	36	81	25	44	30
23	A2						5		fair						0
28	A2				8				fair	81	75	94	60	56	47
30	A2				2			1	good	78	71	87	40	63	17
31	A1					5			fair	75	54	81	90	38	22
32	A3							12	poor	64	57	79	20	38	11
33	A1					2		2	good	81	64	97	90	69	3
37	A1				1		2		good	100	86	100	80	69	4
38	A3						2		fair	100	93	100	85	100	0
39	A1				1		2		good	100	93	100	95	88	7

**Table 5:** Outcomes at one year in patients with at least one parameter poor in the Burwell and Charnley classification (B&C) and/or with pin-tract infection

**Nr**	**Intra/extra articular**	**B&C**	**Pin-tract infection**	**EQ-5D**	**FAOS Pain**	**FAOS Symptom**	**FAOS ADL**	**FAOS Sport**	**FAOS QoL**	**VAS mm**
10	C1	Poor	No	1.000	—	—	—	—	—	8
12	C1	Poor	No	0.727	72	89	90	60	75	31
19	C1	Fair	Yes	0.656	33	61	85	40	63	49
25	C3	Poor	No	0.620	31	21	60	5	0	47
27	C3	Poor	No	0.125	94	57	97	70	63	7
30	A2	Good	Yes	0.796	78	71	87	40	63	17
32	A3	Poor	No	0.767	64	57	79	20	38	11

One patient (# 25) had a residual deformity and developed post-traumatic sympathetic dystrophy which was treated with an ankle joint arthrodesis after 1.5 years. This procedure did not, however, relieve the pain.

**Figure 1:**
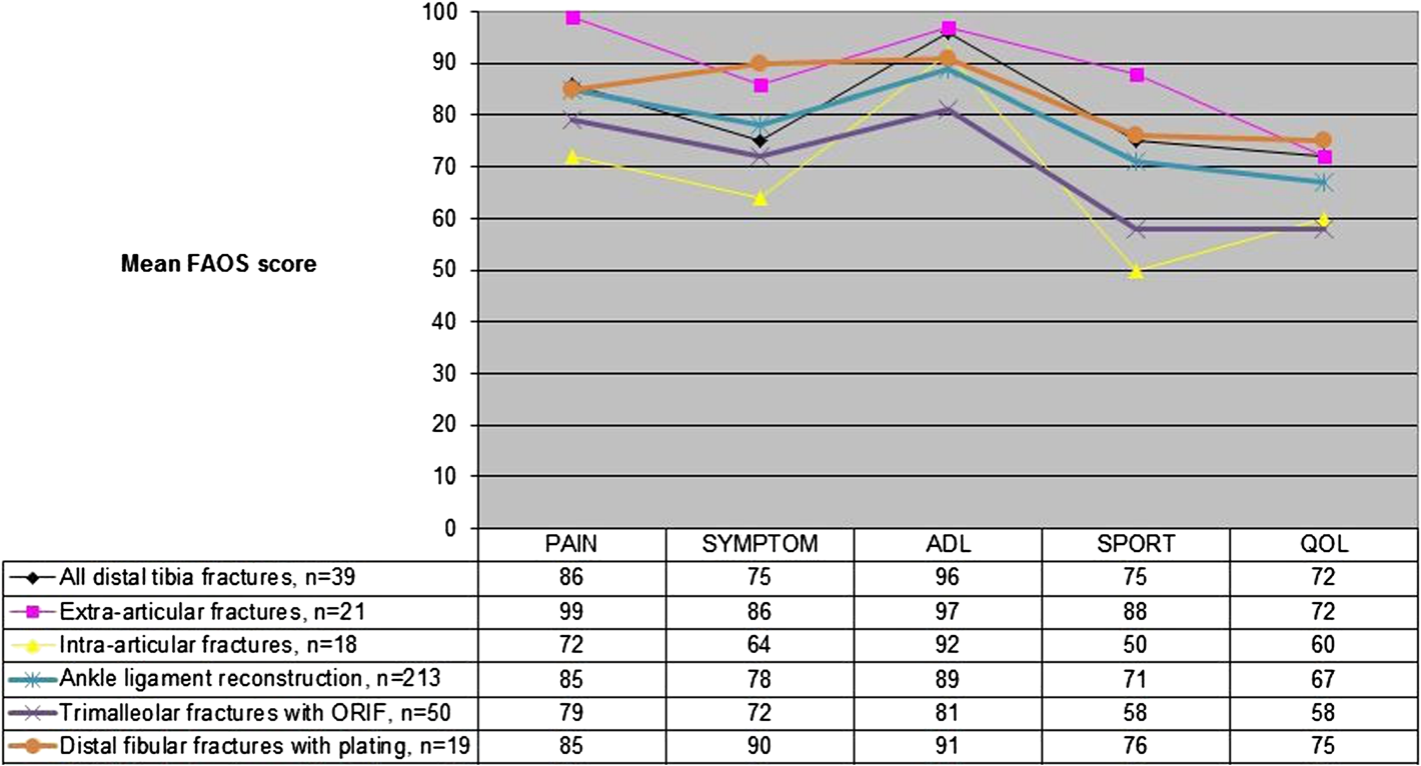
**The FAOS subscores from the present trial compared with ankle ligament reconstruction, trimalleolar and distal fibular fractures**[42]**,**[45]**,**[46].

The pain (VAS), patient satisfaction (VAS), EQ5D, NHP total score at different time intervals and FAOS are shown in Table 6. The pain values (VAS) had reached acceptable levels at 4 weeks but did not improve further between 4 and 12 weeks post-operatively. Patient satisfaction (VAS) with the treatment was generally high in both groups at all follow-up assessments. The NHP total score showed a relatively moderate impact at four and twelve weeks and was almost normal at one year. The EQ-5D values showed a similar pattern. After one year, there were no clinically important differences between the A and C groups in terms of pain (VAS), patient satisfaction (VAS), EQ5D, NHP total score or FAOS. Intra-articular fractures showed a tendency to result in lower FAOS subscores, as shown in Figure 1. The groups were compared with the results from the literature [42,45,46].

**Table 6:** Self-appraisal scores (median and range)

**Median with range**	**Time of assessment**	**Extra-articular fractures**		**Intra-articular fractures**	
**Pain** (VAS)	4 weeks	27	(7–63)	28	(8–58)
	12 weeks	29	(3–56)	26	(0–78)
	1 year	7	(0–54)	14	(0–76)
	At FAOS	6.5	(0–67)	7.5	(0–49)
**Patient satisfaction** (VAS)	4 weeks	14	(1–29)	9	(7–47)
	12 weeks	24	(0–52)	14	(3–35)
	1 year	8	(0–61)	20	(0–53)
	At FAOS	6.5	0(0–67)	7.5	(0–49)
**NHP total**	4 weeks	20.2	(4.9-83.3)	12.4	(0–48.6)
	12 weeks	16.8	(0–69.8)	15.4	(0.-48.7)
	1 year	1.8	(0–76.3)	2.7	(0–39.8)
	At FAOS	1.8	(0–65)	4.1	(0–54)
**EQ5D**	4 weeks	0.62	(−0.07-0.88)	0.52	(0.15-0.73)
	12 weeks	0.69	(0.19-1.0)	0.62	(0.02-0.73)
	1 year	1.0	(0.29-1.0)	0.80	(0.20-1.0)
	At FAOS	0.93	(0.66-1.0)	0.80	(0-36-1.0)

At one year, all the patients had returned to their previous jobs, while sporting activities were severely restricted in the majority of cases (Additional file 1).

## Discussion

The most important finding in this study was the satisfactory outcome of the Ilizarov method, independently of fracture pattern. Using an identical protocol for both intra- and extra-articular fractures, it was possible to operate on all patients without delay, regardless of the status of soft tissues, the size of the distal fragment, and the intra-articular fracture lines or whether a staged protocol should be used. This is also emphasised by the absence of clinically important differences in the present study in terms of the results between the intra- and extra-articular fractures.

In the present study there is a mixture of fractures and from a radiological point of view the treatment of many of these fractures might seem to be uncontroversial.

However, despite the fact that the trauma in a majority of the patients was classified as low energy, most of these patients had other complicating conditions, such as soft-tissue injuries, diaphyseal fracture extension etc. (Table 1). In other words, several of the factors considered when choosing the method of treatment are difficult to evaluate.

Twenty-one of 39 patients in our study had extra-articular fractures, which could possibly have been treated with open reduction and internal fixation using intramedullary nails or plates. However, the use of intramedullary nails in extra-articular distal tibial fractures is technically demanding, because of the widening of the medullary canal in the metaphysis, which raises concern regarding the biomechanical stability and the subsequent increased risk of malunion [47]. The more modern technique with percutaneous angle stable plate fixation is more reliable with a lower rate of complications than previous plate techniques [48-50]. However, until now, both superficial and deep infections have continued to be a problem and there is also a high rate of hardware complications requiring plate removal [51-56]. In this study, thirty patients had a significant diaphyseal extension of the fracture, indicating that a fairly long plate would have to be used.

One common problem with external fixators of all types is the risk of skin-penetrating infections (pin site and pin tract infections). The incidence of pin site infections reported in the literature varies from 4.5% to 71% [57] and pin tract infection varies from 10% to 50% [58]. Parameswaran et al. [59], found that ring fixators had the lowest incidence of infection compared with unilateral and hybrid fixators. Pin site infections were frequent in the present study; however, they did not constitute a major problem, as all the infections healed following minor intervention. In the present study, 3.7% of the pin sites became infected, while only two patients had more severe pin tract infections.

Ristiniemi used hybrid ring fixators in different types of intra-articular fracture, with or without osteoinduction [60]. In the group without osteoinduction, the healing period was 21 weeks, which compares favorably with the 15 weeks for similar fractures in our study.

The amount of residual deformity that can be accepted is still controversial [43]. It is difficult to correlate the postoperative radiological findings to the clinical result and to use this as a prognostic factor. In a 10-year follow-up, Etter and Ganz [61] retrospectively examined how the fracture pattern and quality of reduction correlated to postoperative arthritis in 41 patients with plafond fractures treated with internal fixation. Anatomical reduction was correlated to a better prognosis in terms of a lower risk of post-traumatic osteoarthritis, but it did not guarantee a good clinical result. Severe osteoarthritis present at late follow-up did not correlate with poor subjective or objective function. DeCoster et al. [62] came to the same conclusions using the rank order method to assess the quality of articular reduction in the outcome of displaced intra-articular distal tibia fractures in 25 patients treated with articulated external fixation and limited internal fixation. With ten B3, three C1, ten C2 and twelve C3 fractures, Marsh et al. [63] rated the quality of reduction as good in 14 ankles, fair in 15 and poor in 6, using the same radiological evaluation method as in the present study. They did not find any association between the fracture type and the clinical outcome measures. In their study, the majority of the patients had some limitation with regard to recreational activities, with an inability to run as the most common complaint. Fourteen patients had to change jobs due to the ankle injury.

Williams et al. [64] determined which fracture- and patient-specific variables affected the outcome most in 29 patients with tibial plafond fractures. They evaluated their patients a minimum of two years from the time of the injury. Outcome was assessed by four independent measures; radiographic osteoarthritis score, subjective ankle score, the Short Form-36 (SF-36), and the patient’s ability to return to work. Interestingly, the four outcomes did not correlate with one another. They also found that the ability to return to work was affected by the patient’s level of education and highlighted the difficulties of predicting patient outcome in, these severe articular fractures.

Pollak et al. [65] evaluated eighty patients, treated with bridging external fixation and/or internal fixation, at a mean of 3.2 years after injury. They analysed general health, walking ability, range of motion, pain, and stair-climbing as well employment status. Their general conclusion was that pilon fractures could have persistent and devastating consequences for patient-health and well-being. In approximately 30% of their patients, the injury prevented a return to work.

In the present study, several self-appraisals were used in our study, both general (NHP and EQ-5D) and more specific (Pain in the affected limb -VAS and FAOS). The patients with metaphyseal fractures without joint engagement were in an almost normal situation at one year postoperatively, but the intra-articular fractures were also better than the reported by the patients in the above mentioned studies. Despite successful treatment and improvement in their outcomes, the FAOS subscores showed the lowest values for Sports and QoL activities especially in the C fractures. Apart from this, they did not differ significantly, compared with patients after operated ankle ligaments, trimalleolar or distal fibular fractures [42,45,46]. All the patients returned to work while sporting activities were severely restricted in both groups without significant differences, but we observed a trend towards more seriously affected Sports and QoL for the group with extra-articular fractures.

The results of the follow-up observed in this study might differ in the future in terms of functional outcome. Marsh et al. [63] claimed that, although tibial plafond fractures have a negative effect on ankle function and pain, at a minimum of five years after the injury, few patients required secondary reconstructive procedures because these symptoms tend to decrease during a long time period after the acute injury.

## Conclusions

The study shows that it is possible to achieve a satisfactory outcome, in distal metaphyseal tibia fractures, with the Ilizarov technique allowing early definitive treatment and unrestricted weight-bearing. The fractures were treated immediately after the injury, regardless of soft-tissue damage. This was done with a similar low complication rate in both the extra-articular and the intra-articular fractures. Patient compliance was good. The residual deformities were within the range in which the risk of developing post-traumatic osteoarthritis can be expected to be low.

## Abbreviations

AO: Arbeitsgemeinschaft für Osteosynthesefragen; ORIF: Open Reduction Internal Fixation.

## Competing interests

The authors declare that they have no competing interests.

## Authors’ contributions

TR conducted the study, treated the patients and wrote the manuscript. JK and LN participated in the design of the study which LN supervised. They both helped to analyse the results and revised the manuscript together with BE. All the authors agreed on the final content of the manuscript.

## Pre-publication history

The pre-publication history for this paper can be accessed here:

http://www.biomedcentral.com/1471-2474/14/30/prepub

## Supplementary Material

Additional file 1: Table S1 Details of all patients treated with Ilizarov application and fracture types; **Table S2.** The outcomes in the two subgroups with A fractures and C fractures respectively; **Table S3.** Complications with all fractures; **Table S4.** Functional outcome at one year follow-up comparing the range of motion between subgroups with A fractures and C fractures; **Table S5.** The radiological outcome in patients with at least one parameter fair and poor in the Burwell and Charnley classification analyzed with FAOS and VAS satisfaction in both groups at the one-year control; **Table S6.** The radiological outcomes in patients with at least one parameter poor in the Burwell and Charnley classification analyzed with pin-tract infection, EQ-5D, FAOS and VAS satisfaction at the one-year control; **Table S7.** The outcome according with patients’ self-appraisal controls in the two subgroups with extra-articular (A fractures) and intra-articular (C fractures) fractures; **Table S8.** The outcome according with patients’ self-appraisal controls in the two subgroups with extra-articular (A-type) and intra-articular (C type) fractures when FAOS were done; **Figure S1.** The FAOS subscores from the present trial compared with ankle ligament reconstruction, trimalleolar and distal fibular fractures [42,45,46]. Click here for file
