# Supplementary material for: Treatment of distal tibial fractures with the Ilizarov external fixator - a prospective observational study in 39 consecutive patients
Source: BMC Musculoskelet Disord. 2013 Jan 17;14:30. doi: 10.1186/1471-2474-14-30 (PMC3626620; doi:10.1186/1471-2474-14-30)
Supplement: Additional file 1: Table S1 — Details of all patients treated with Ilizarov application and fracture types; Table S2. The outcomes in the two subgroups with A fractures and C fractures respectively; Table S3. Complications with all fractures; Table S4. Functional outcome at one year follow-up comparing the range of motion between subgroups with A fractures and C fractures; Table S5. The radiological outcome in patients with at least one parameter fair and poor in the Burwell and Charnley classification analyzed with FAOS and VAS satisfaction in both groups at the one-year control; Table S6. The radiological outcomes in patients with at least one parameter poor in the Burwell and Charnley classification analyzed with pin-tract infection, EQ-5D, FAOS and VAS satisfaction at the one-year control; Table S7. The outcome according with patients’ self-appraisal controls in the two subgroups with extra-articular (A fractures) and intra-articular (C fractures) fractures; Table S8. The outcome according with patients’ self-appraisal controls in the two subgroups with extra-articular (A-type) and intra-articular (C type) fractures when FAOS were done; Figure S1. The FAOS subscores from the present trial compared with ankle ligament reconstruction, trimalleolar and distal fibular fractures [42,45,46]. [file 1471-2474-14-30-S1.doc]

**Table 1** Details of all patients treated with Ilizarov application and fracture types

Case Age Mechanism of Injury Rüedi-Allgöwer AO

1 20 Fall 0 A2

2 62 Fall 0 A2

3 57 Riding I C1

4 59 Fall 0 A2

5 39 RTA II C1

6 55 Work 0 A2

7 56 Fall 0 A2

8 39 Fall 0 A2

9 33 RTA 0 A3

10 59 Fall I C1

11 70 Fall 0 A2

12 61 Work I C1

13 58 Fall 0 A3

14 50 Fall II C3

15 46 Fall 0 A1

16 43 Fall 0 A3

17 29 RTA 0 A3

18 50 RTA II C1

19 46 Fall I C1

20 39 Fall 0 A2

21 63 Fall 0 A2

22 42 Fall I C1

23 21 Fall 0 A2

24 42 Fall III C3

25 66 Fall III C2

26 51 Fall I C1

27 62 Fall III C1

28 54 RTA I C1

29 54 Fall I C1

30 68 Fall 0 A2

31 56 Fall 0 A1

32 43 Skiing 0 A3

33 44 Skiing 0 A1

34 48 Fall I C1

35 56 RTA 0 A3

36 24 Fall II C1

37 42 Fall 0 A1

38 70 Fall 0 A3

39 49 Fall 0 A1

RTA road traffic accident

**Table 2** The outcomes in the two subgroups with A fractures and C fractures respectively.

A Fractures C Fractures

n=23 n=16

median 95% CI median 95% CI

Surgery delay (days) 2 1-3 1 1-2

Operation time (min) 152 127-160 178 145-221

Hospital stay (days) 5 4-6 5 4-6

External fixation (weeks) 17 7-21 15 12-19

**Table 3** Complications with all fractures.

Complications n=39

Compartment syndrome 0

DVT 0

Secondary dislocation 2

Pin site infections 41

Pin track infection 2

Osteomyelitis with radiological signs 0

Sympathetic dystrophy 1

Nerve injury 0

**Table 4** Functional outcome at one year follow-up comparing the range of motion between subgroups with A fractures and C fractures

|  | A Fractures (n=23) | | C Fractures (n=16) | |  |
| --- | --- | --- | --- | --- | --- |
|  | uninjured | injured | uninjured | injured | p-value^1^ |
| Ankle dorsiflexion | 20° (16-220°) | 18° (12-20°) | 21° (16-24°) | 16.5° (14-20°) | 0.86 |
| Ankle plantarflexion | 37° (28-40°) | 30° (20-38°) | 35° (32-46°) | 20° (12-32°) | 0.08 |

^1^ Comparison between the groups with respect to the injured side by Mann-Whitney test. Median values and confidence intervals are presented.

**Table 5**  The radiological outcome in patients with at least one parameter fair and poor in the Burwell and Charnley classification analyzed with FAOS and VAS satisfaction in both groups at the one-year control.

| A | | | Central | Talar | Mortise | Vr | Vl | Anterior | Posterior | FAOS | FAOS | FAOS | FAOS | FAOS | VAS |
| --- | --- | --- | --- | --- | --- | --- | --- | --- | --- | --- | --- | --- | --- | --- | --- |
| Fract | | fragment  (mm) | | subluxation (mm) | widening  (mm) | ° | ° | ° | ° | Pain | Symptom | ADL | Sport | QoL | mm |
|  | |  | |  |  |  |  |  |  |  |  |  |  |  |  |
| 1 | | <2 | | <0.5 | <0.5 | 0 | 0 | **7** | 0 | — | — | — | — | — | 0 |
| 4 | | <2 | | <0.5 | <0.5 | 0 | 4 | 0 | **8** | 100 | 100 | 100 | 100 | 100 | 18 |
| 6 | | <2 | | <0.5 | <0.5 | 0 | **8** | 1 | 0 | 83 | 68 | 93 | 70 | 75 | 0 |
| 7 | | <2 | | <0.5 | <0.5 | 0 | 0 | 0 | **8** | — | — | — | — | — | 26 |
| 9 | | <2 | | <0.5 | <0.5 | 0 | **5** | **5** | 0 | — | — | — | — | — | 0 |
| 13 | | <2 | | <0.5 | <0.5 | 0 | **5** | **4** | 0 | 97 | 86 | 96 | 100 | 94 | 38 |
| 15 | | <2 | | <0.5 | <0.5 | **5** | 0 | **8** | 0 | 100 | 86 | 100 | 100 | 100 | 0 |
| 19 | | <2 | | <0.5 | <0.5 | 0 | 3 | 0 | **7** | 61 | 61 | 85 | 40 | 63 | 49 |
| 21 | | <2 | | <0.5 | <0.5 | **6** | 0 | 4 | 0 | 100 | 100 | 100 | 100 | 100 | 7 |
| 23 | | <2 | | <0.5 | <0.5 | 0 | 0 | 5 | 0 | — | — | — | — | — | 0 |
| 28 | | <2 | | <0.5 | <0.5 | **8** | 0 | 0 | 0 | 81 | 75 | 94 | 60 | 56 | 47 |
| 31 | | <2 | | <0.5 | <0.5 | 0 | **5** | 0 | 0 | 75 | 54 | 81 | 90 | 38 | 22 |
| ***32*** | | <2 | | <0.5 | <0.5 | 0 | 0 | 0 | ***12*** | 64 | 57 | 79 | 20 | 38 | 11 |
| 38 | | <2 | | <0.5 | <0.5 | 0 | 0 | **7** | 0 | 100 | 93 | 100 | 85 | 100 | 0 |
| | C  Fract | | | --- | --- | |  |  | | | |  |  |  |  |  |  |  |  |  |  |  |  |  |
| 2 | <2 | | | <0.5 | <0.5 | **9** | 0 | 0 | **5** | — | — | — | — | — | 0 |
| 3 | <2 | | | <0.5 | <0.5 | 0 | 0 | 0 | **7** | 100 | 93 | 100 | 100 | 94 | 7 |
| 5 | <2 | | | <0.5 | <0.5 | **8** | 0 | 0 | 4 | 72 | 21 | 93 | 35 | 38 | 18 |
| ***10*** | <2 | | | <0.5 | <0.5 | 0 | ***10*** | ***10*** | 0 | — | — | — | — | — | 1 |
| ***12*** | <2 | | | <0.5 | <0.5 | ***11*** | 0 | 2 | 0 | 72 | 89 | 90 | 60 | 75 | 31 |
| 14 | <2 | | | <0.5 | <0.5 | **8** | 0 | 0 | 0 | 92 | 86 | 96 | 100 | 88 | 10 |
| 24 | <2 | | | <0.5 | <0.5 | 0 | **9** | **6** | 0 | 33 | 14 | 71 | 0 | 19 | 0 |
| ***25*** | <2 | | | <0.5 | <0.5 | 0 | ***12*** | 0 | 3 | 31 | 21 | 60 | 5 | 0 | 47 |
| 26 | <2 | | | <0.5 | <0.5 | 0 | **6** | 3 | 0 | 39 | 64 | 72 | 5 | 75 | 76 |
| ***27*** | **9** | | | **6** | 5 | 0 | ***16*** | 0 | 1 | 94 | 57 | 97 | 70 | 63 | 7 |
| 34 | <2 | | | <0.5 | <0.5 | **8** | 0 | 2 | 0 | 67 | 54 | 85 | 40 | 31 | 41 |

**Table 6**  The radiological outcomes in patients with at least one parameter poor in the Burwell and Charnley classification analyzed with pin-tract infection, EQ-5D, FAOS and VAS satisfaction at the one-year control

| **Nr** | **Intra/extra**  **articular** | **Rx**  **B&C** | **Pin-tract**  **infection** | **EQ-5D** | **FAOS Pain** | **FAOS Symptom** | **FAOS ADL** | **FAOS Sport** | **FAOS QoL** | **VAS mm** |
| --- | --- | --- | --- | --- | --- | --- | --- | --- | --- | --- |
| 10 | C1 | poor | No | 1.000 | — | — | — | — | — | 1 |
| 12 | C1 | poor | No | 0.727 | 72 | 89 | 90 | 60 | 75 | 31 |
| 19 | C1 | fair | Yes | 0.656 |  |  |  |  |  | 49 |
| 25 | C3 | poor | No | 0.620 | 31 | 21 | 60 | 5 | 0 | 47 |
| 27 | C3 | poor | No | 0.125 | 94 | 57 | 97 | 70 | 63 | 7 |
| 30 | A2 | good | Yes | 0.796 |  |  |  |  |  | 17 |
| 32 | A3 | poor | No | 0.767 | 64 | 57 | 79 | 20 | 38 | 11 |

|  |  |  |  |  |  |  |  |  |  |  |  |  |  |
| --- | --- | --- | --- | --- | --- | --- | --- | --- | --- | --- | --- | --- | --- |

|  |  |  |  |  |
| --- | --- | --- | --- | --- |
|  |  |  |  |  |
|  |  |  |  |  |
|  |  |  |  |  |
|  |  |  |  |  |

**Table 7** The outcome according with patients’ self-appraisal controls in the two subgroups with extra-articular (A fractures) and intra-articular (C fractures) fractures

**Median (95 % CI)**

|  | | **Time of assessment** | **A Fractures** | **C Fractures** | |
| --- | --- | --- | --- | --- | --- |
|  | |  |  |  | |
| **Pain (VAS)** | | 4 weeks | 27 (18-29) | 28 (8-58) | |
|  | | 12 weeks | 33.5 (18-46) | 23 (6-38) | |
|  | | 1 year | 7 (0 -26) | 9 (7-38) | |
|  | |  |  |  | |
| **Patient satisfaction (VAS)** | | 4 weeks | 14 (8-27) | 9 (8-47) | |
|  | | 12 weeks | 25.5 (10-31) | 12 (7-26) | |
|  | | 1 year | 8 (0-17) | 20 (8-28) | |
|  | |  |  |  | |
| **NHP total** | | 4 weeks | 20.2 (13.9-32.2) | 12.4 (6-23.2) | |
|  | | 12 weeks | 16.8 (0-69.8) | 15.4 (0-36.6) | |
|  | | 1 year | 2.4 (0-8.4) | 1.8 (0-8.8) | |
|  | |  |  |  | |
| **EQ5D** | | 4 weeks | 0.59 (0.36-0.69) | 0.62 (0.36-0.69) | |
|  | | 12 weeks | 0.69 (0.36-0.69) | 0.69 (0.36-0.69) | |
|  | | 1 year | 0.93 (0.66-1.0) | 0.80 (0.69-1.0) | |
|  |  | |  |  |  |
|  |  | |  |  |  |
|  |  | |  |  |  |
|  |  | |  |  |  |
|  |  | |  |  |  |
|  |  | |  |  |  |

There were no statistically significant differences between the groups with respect to the parameters in the table but there was a tendency for Patient satisfaction at 1 year (p-value=0.08) and NHP at 4 weeks (p-value=0.09).

.

**Table 8** The outcome according with patients’ self-appraisal controls in the two subgroups with extra-articular (A-type) and intra-articular (C type) fractures when FAOS were done.

**Median (95% CI)**

A Fractures C Fractures

Pain (VAS) 6.5 (0-28) 7.5 (0-39)

Patient satisfaction (VAS) 7 (0-17) 8 (1-39)

NHP total 1.8 (0-5.6) 4.1 (0-16.5)

EQ5-D 0.93 (0.8-1.0) 0.80 (0.69-1.0)

**Figure 1** The FAOS subscores from the present trial compared with ankle ligament reconstruction at 12 years*


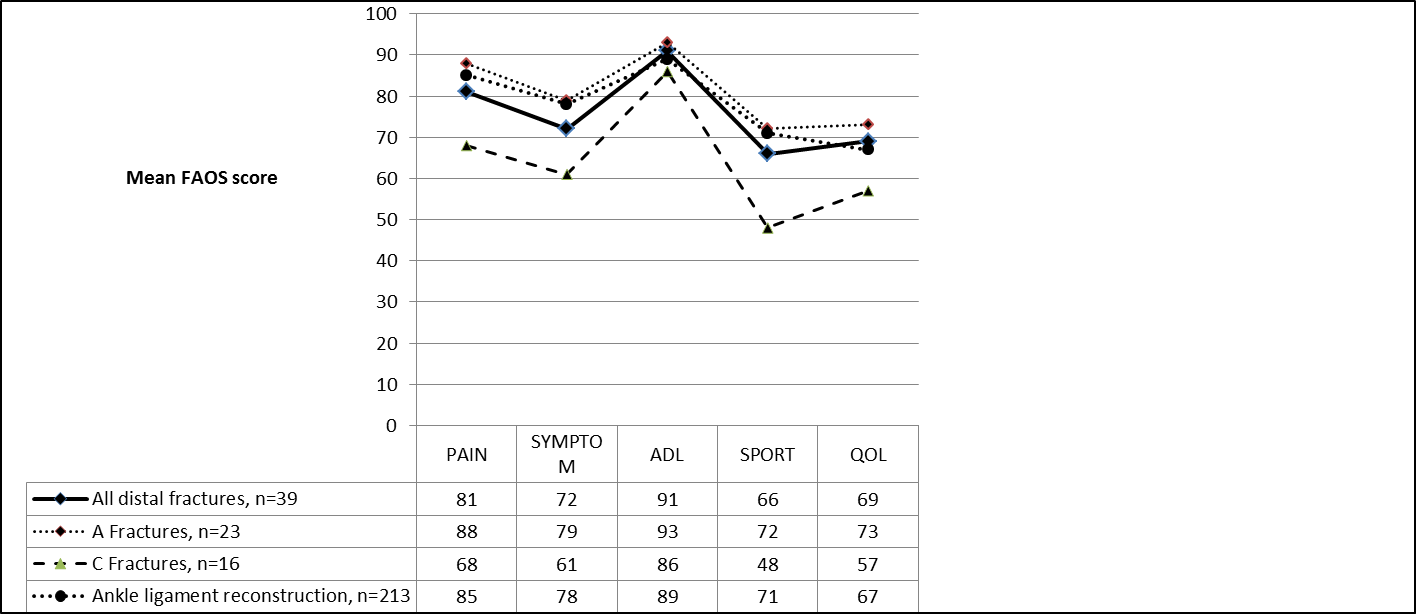


* Roos EM, Brandsson S, Karlsson J: **Validation of the Foot and Ankle Outcome Score for Ankle Ligament Reconstruction.** *Foot Ankle Int* 2001, **22-10:** 788-94.
